# Action of Air and Water on Lead

**Published:** 1830-04-01

**Authors:** 


### XXIII.

### ACTION OF AIR AND PURE WATER ON LEAD.

Dr. Christison, in his admirable work on poisons, has dedicated a section to a consideration of the various ways in which lead is apt to be introduced into the body, by the action of chemical agents on the metal itself.

It is well known that lead is tarnished by exposure to air. This does not arise, as some have supposed, from oxidation, but from a thin crust of carbonate of lead being formed, as may be proved by scraping off the crust and immersing it in acetic acid, when it will be dissolved with a brisk effervescence. The formation of this crust is accelerated by moisture, and probably by the presence of extra-carbonic acid in the air.*

The action of water on lead, which is of much greater consequence, has been observed and recorded since the days of Vitruvius, the Roman architect, who forbade the use of lead for conduits of water, because cerusse (he says) is formed on it. Galen also condemns the use of lead pipes. In our own times Dr. Lambe inferred, from a number of experiments, that most, if not all spring waters possess the power of corroding and dissolving lead to such an extent as to be rendered unfit for the use of man, and that this solvent power is imparted to them by some of their saline ingredients. Guyton-Morveau proved that distilled water acts rapidly on lead, by converting it into a hydrated oxide, and that some natural waters, which hardly attack lead at all, are prevented doing so by the salts they

* In a late sitting of the Westminster Medical Society, Dr. Thomson stated, from a number of experiments and observations, that none of the salts of lead, with the exception of the carbonate, are poisonous. Where mischief had resulted from the taking of acetate and subacetate of lead, he believed the cause was the conversion of these into carbonate.

hold in solution. Still more recently Dr. Thomson, of Glasgow, maintained that the lead in waters was not in a state of solution, but only suspension; and believed the quantity in waters which have passed through leaden pipes, &c. to be too inconsiderable to have any deleterious effect on man's health. Dr. Christison determined, therefore, to investigate the subject anew.

"Distilled water, deprived of its gases by ebullition, and excluded from contact with the air, has no action whatever on lead. If the water contains the customary gases in solution, the surface of the metal, freshly polished, becomes rapidly dull and white. But if the surface of the water be not at the same time exposed to the air, the action soon comes to a close. When the air, on the other hand, is allowed free access to the water, a white powder appears in a few minutes around the lead; and this goes on increasing till in the course of a few days there is formed a large quantity of white, pearly scales, which partly float in the water, but are chiefly deposited on the bottom of the vessel. In twelve ounces of distilled water, contained in a shallow glass basin, loosely covered to exclude the dust, twelve brightly polished lead rods, weighing 340 grains, will lose two grains and a half in eight days; and the lead will then shew evident marks of corrosion. The process of corrosion goes on so long as atmospheric air is allowed to play freely on the surface of the water, but gradually becomes less and less, provided the water be not occasionally shaken, to prevent the adhesion of the powder to the surface of the lead."

During these changes, a minute quantity of lead is dissolved, as proved by filtration, acidulation, and evaporation. This lead, dissolved by the aerated water, is in the form of carbonate. It dissolves with brisk effervescence in acetic acid, becomes yellow when heated to redness, and the loss of weight then sustained by it corresponds with the atomic proportions assigned for the carbonate of lead.

The solving property of pure aerated water on lead is variously modified by foreign ingredients held in solution. Of these, none are more remarkable than the neutral salts, which all impair the corrosive power of the water; for instance, the sulphates, muriates, carbonates, hydriodates, nitrates, acetates, tartrates, arseniates—which are all the neutral salts Dr. C. has tried. The degree of this anti-corrosive power differs much in different salts. The acetate of soda is but an imperfect preventative; on the contrary, arseniate of soda is a complete preservative. Phosphate of soda and hydriodate of potass are nearly effectual preservatives. Muriate of soda and sulphate of lime hold a middle place between these extremes, and are both of them much more powerful preservatives than Guyton-Morveau imagined. The preservative power depends not on the base, but the acid of the salt. Those whose acid forms with lead a soluble salt are least energetic, and *vice versâ*.

"When the protection afforded is complete, as for example by a 27,000th of phosphate of soda, a 12,000th of arseniate of soda, or a 4000th of sulphate of soda, the lead undergoes no change in appearance or in weight for several hours, or even days. At length the surface becomes dull, then white, and gradually a uniform film is formed over it. This film, examined at an early period, is found to consist of carbonate of lead,—being entirely soluble in diluted acetic acid, although the salt in solution is a sulphate or phosphate. But after a few weeks the carbonate is mixed with a salt of lead, containing the acid of a part of the neutral salt dissolved in the water: If, after five or six weeks' immersion in a preservative solution of phosphate or sulphate of soda, the film on the lead be scraped off and immersed in diluted acetic acid, effervescence and solution take place, but a part of the powder remains undissolved; and if the protecting salt has been the muriate of soda, the whole powder is dissolved, but muriatic acid will be found in solution by its proper test, the nitrate of silver.—In all such protecting solutions the lead gains weight for some weeks; but at length it ceases to undergo farther change, and is not even acted on if removed into distilled water. The crust, when formed thus slowly, adheres with great firmness. The most careful analysis cannot detect any lead, either dissolved in the water, or floating in it, or united with the insoluble matter left on the side of the glass by evaporation. In short, the preservation of the lead from corrosion, and of the water from impregnation with lead, is complete."

Dr. Lambe was of opinion that rain water did not corrode lead; but this is not quite correct. Rain or snow water, before it touches the earth, is nearly as pure as distilled water, and acts with nearly as much rapidity on the lead. But when rain or snow water is collected in a great city, and, consequently, contaminated with various principles, its activity is much impaired. The eaves-droppings from Dr. Christison's house, after a fall of rain succeeding to a long drought, was almost entirely inactive on lead;—but, after 12 or 24 hours' rain, (when the gutters, &c. are well washed by the current) the water corrodes as rapidly as distilled water.

"Hence, perhaps, even in a town, but at all events certainly in the country, it would be wrong to use for culinary purposes rain or snow-water which has run from lead roofs or spouts recently erected. When the roof or spout has been exposed for some time to the weather the danger is of course much lessened, if not entirely removed; because exposure to the weather encrusts it with a firmly adhering coat of carbonate, through which, as already observed, even distilled water will not act. But I believe it would be right to condemn the turning even old leaden roofs to the purpose of collecting water for the kitchen. Although the purest rain water cannot act on them when it is once fairly at repose, we do not know what may be the effect of the impetus of the falling rain on the crust of carbonate; and if the crust should happen to be thus worn considerably, or detached by more obvious accidents, the corrosion would then go on with rapidity as long as the shower lasted."

Most spring waters, unlike rain or snow water, have little or no action on lead, because they generally contain a considerable proportion of muriates and sulphates. The water of Edinburgh appears to be nearly destitute of all action on lead;—and we think the good citizens of London need not be much afraid of the painter's colic while they are supplied with water from the Thames, or even the New River. If purity of water be a dangerous property, the metropolis is as secure as if they drank nothing but nectar.

---

